# Effects of a 6-Week Hip and Ankle Mobility-Based Rehabilitation Program on Clinical, Neuromuscular, and Functional Outcomes in Male Collegiate Athletes with Patellofemoral Pain: A Randomized Controlled Trial

**DOI:** 10.3390/life16061013

**Published:** 2026-06-17

**Authors:** Hengquan Xu, Zhaozhi Feng, Yue Dou, Gang Wang

**Affiliations:** 1 School of Sport and Health Science, Xi’an Physical Education University, Xi’an 710068, China; 17789275685@163.com (H.X.);; 2 China Football College, Beijing Sport University, Beijing 100084, China

**Keywords:** anterior knee pain, kinetic chain, exercise therapy, surface electromyography, dynamic balance

## Abstract

Patellofemoral pain (PFP) in athletes is associated with lower-limb kinetic-chain constraints, yet rehabilitation strategies targeting both hip and ankle mobility remain insufficiently examined. This assessor-blinded randomized controlled trial investigated the effects of a 6-week hip and ankle mobility-based rehabilitation program in male collegiate athletes with PFP. Forty-eight participants were assigned using computer-generated 1:1 randomization to an intervention group (*n* = 24) or a control group (*n* = 24). The intervention group completed supervised hip and ankle mobility rehabilitation three times weekly, whereas the control group maintained regular sport-specific training only. Co-primary outcomes were pain intensity assessed using a 10-cm visual analog scale (VAS) and knee-related function assessed using the Kujala score. Secondary outcomes included hip rotation range of motion, weight-bearing ankle dorsiflexion, vastus medialis–vastus lateralis (VM–VL) onset timing, Y-Balance Test (YBT) composite score, and countermovement jump (CMJ) height. Significant group × time interactions favored the intervention group for VAS (*p* < 0.0001; partial η^2^ = 0.436; change difference: −1.54 cm; 95% CI: −2.06 to −1.02) and Kujala score (*p* < 0.0001; partial η^2^ = 0.285; change difference: 8.00 points; 95% CI: 4.24 to 11.76). Significant interactions were also observed for hip internal and external rotation range of motion, weight-bearing ankle dorsiflexion, VM–VL onset timing during a controlled squat task, and YBT composite score (all *p* ≤ 0.0405; partial η^2^ = 0.088–0.374). No significant group × time interaction was observed for CMJ height (*p* = 0.0511; partial η^2^ = 0.080). These findings suggest that, compared with regular sport-specific training alone, adding a supervised hip and ankle mobility-based rehabilitation program may improve pain, knee-related function, targeted mobility outcomes, VM–VL onset timing during a controlled squat task, and dynamic balance in the short term. However, because the control group did not receive an active or attention-matched intervention, these findings should be interpreted as the added effect of the supervised rehabilitation program rather than as definitive evidence of mobility-specific treatment effects. In addition, because patellar tracking, knee kinematics, joint kinetics, and patellofemoral joint loading were not directly measured, the findings should be interpreted as clinical and functional outcome changes rather than direct evidence of a confirmed biomechanical mechanism. Trial registration: NCT07542236.

## 1. Introduction

Patellofemoral pain (PFP) is a common overuse-related knee condition among physically active individuals and athletes [1], typically presenting as peripatellar or retropatellar pain during activities that increase patellofemoral joint loading, such as squatting, stair ascent and descent, running, jumping, and prolonged sitting [2]. Although PFP is often regarded as a non-traumatic and relatively common musculoskeletal pain condition, its clinical relevance in athletic populations should not be underestimated. For athletes, knee pain is not merely a subjective symptom; it may disrupt training continuity, alter lower-limb movement strategies, reduce tolerance to repeated loading, and impair sport-related functional performance [3]. This issue is particularly important for collegiate athletes, who are often required to maintain regular sport-specific training while repeatedly performing high-load lower-limb tasks, including running, jumping, landing, support, and squatting. Therefore, effective rehabilitation for PFP in athletes should not focus solely on pain relief, but should also address the restoration of functional movement capacity and sustained sport participation [4].

Exercise therapy is widely recognized as a core component of conservative management for PFP [5], and existing evidence strongly supports an intervention approach that considers the lower limb as an integrated functional system rather than focusing solely on the knee joint. Patellofemoral pain is currently regarded as a multifactorial condition rather than a disorder explained by a single biomechanical impairment. Contemporary rehabilitation recommendations emphasize exercise therapy and education as core components, often combined with patient-tailored strategies such as hip- and knee-focused strengthening, neuromuscular training, movement or running retraining, foot orthoses, manual therapy, or taping when clinically indicated [4,6]. Within this broader framework, hip and ankle mobility restrictions should not be interpreted as the sole cause of PFP, but rather as potentially modifiable kinetic-chain constraints that may interact with strength, movement control, training load, and symptom-related adaptations [7]. Therefore, examining whether a mobility-focused program can improve clinical and functional outcomes may help clarify the specific contribution of mobility restoration within a broader multimodal rehabilitation context. In particular, rehabilitation programs combining hip- and knee-focused exercises have been shown to improve pain and self-reported function, highlighting the clinical importance of proximal control and lower-limb mechanical factors in PFP rehabilitation. However, for athletes who are continuously exposed to high training loads, the central question is no longer simply whether exercise therapy is effective, but rather which modifiable biomechanical constraints should be prioritized under sustained high-load sport demands to maximize functional recovery while relieving symptoms. The current literature has largely focused on strengthening or movement-control training [8,9], whereas fundamental mobility restrictions within the lower-limb kinetic chain—particularly limitations in hip and ankle mobility—have received comparatively limited attention as primary targets within structured rehabilitation. This gap is especially relevant for athletes: even in the absence of marked strength deficits, restricted hip and ankle mobility may force compensatory movement strategies during repeated squatting, jumping, landing, and running tasks, thereby reducing load-absorption efficiency and increasing patellofemoral joint stress [10,11]. Further investigation is therefore needed to determine whether a rehabilitation strategy centered on reducing proximal hip and distal ankle mobility constraints can provide additional clinical and functional benefits for athletes with PFP beyond regular sport-specific training.

From a lower-limb kinetic-chain perspective, hip and ankle mobility are not isolated local anatomical measures, but key constraints that influence load distribution across the hip–knee–ankle complex during functional tasks such as running, jumping, and squatting [12]. Adequate hip rotational mobility provides the femur with the necessary capacity for adaptation and shock absorption during multiplanar movements, including squatting, landing, and changing direction. When this mobility is restricted, athletes may have fewer effective movement strategies for maintaining lower-limb alignment and pelvic stability [13]. Similarly, sufficient weight-bearing ankle dorsiflexion is fundamental for tibial progression during closed-chain tasks. When dorsiflexion is limited, the body may compensate through excessive foot motion, such as pronation, increased frontal-plane knee motion, such as dynamic knee valgus, or altered trunk and hip strategies [14]. These compensatory patterns may not only increase mechanical stress on the patellofemoral joint, but also reduce lower-limb impact-absorption efficiency during repeated high-load tasks [15]. Importantly, such mechanical constraints may also interfere with neuromuscular coordination. Restricted movement options across the hip–knee–ankle complex may also be associated with altered quadriceps activation timing, including differences in the relative onset timing of the vastus medialis (VM) and vastus lateralis (VL). However, VM–VL onset timing should be regarded as an indirect neuromuscular measure rather than a direct indicator of patellar tracking, joint loading, or knee joint mechanics [16]. Therefore, targeted improvements in hip rotation and weight-bearing ankle dorsiflexion may represent a clinically relevant strategy for addressing both biomechanical dysfunction and neuromuscular coordination deficits in PFP.

Although hip and ankle mobility constraints have a strong theoretical basis in PFP management, three key gaps remain in the clinical intervention literature. First, most exercise-based rehabilitation studies for PFP have focused on strengthening and movement-control approaches, whereas few randomized controlled trials have independently examined hip rotational mobility and weight-bearing ankle dorsiflexion as central targets within a structured rehabilitation program [17]. Second, although pain relief and self-reported functional improvement are important clinical endpoints, subjective scales alone cannot clarify whether an intervention modifies the underlying biomechanical and neuromuscular impairments that may contribute to persistent symptoms [18]. Third, clinical evidence in competitive athletic populations remains limited, particularly among collegiate athletes who must maintain regular sport-specific training during rehabilitation and continue to experience repeated exposure to high lower-limb impact loads. Therefore, a more multidimensional research question needs to be addressed in this population: can a rehabilitation program centered on reducing hip and ankle mobility restrictions alleviate pain while also improving direct intervention targets, quadriceps coordination, and functional movement capacity? Accordingly, a randomized controlled trial using a multilayered outcome framework is warranted to systematically evaluate the clinical efficacy, biomechanical responses, neuromuscular adaptations, and functional transfer of this mobility-based rehabilitation strategy.

In addition to clinical symptoms, targeted mobility outcomes, neuromuscular coordination, and dynamic balance, countermovement jump (CMJ) performance was included as an exploratory functional transfer outcome. CMJ was selected because it is a commonly used lower-limb functional performance task that requires coordinated hip–knee–ankle contribution during rapid force production. In the context of this mobility-based rehabilitation program, CMJ was used to examine whether improvements in hip rotational mobility, weight-bearing ankle dorsiflexion, and lower-limb movement control could transfer to an explosive functional task. However, because the intervention did not include specific strength, power, or plyometric training, CMJ was not considered a primary expected adaptation.

Therefore, this randomized controlled trial aimed to investigate the effects of a 6-week hip and ankle mobility-based rehabilitation program on pain intensity, knee-related function, targeted mobility outcomes, neuromuscular coordination, dynamic balance, and exploratory explosive lower-limb performance in male collegiate athletes with PFP. We hypothesized that, compared with maintaining regular sport-specific training alone, this intervention would produce greater improvements in pain and knee-related function and would be accompanied by improvements in hip rotational mobility, weight-bearing ankle dorsiflexion, VM–VL activation synchrony, and dynamic balance. CMJ performance was analyzed as an exploratory outcome.

## 2. Materials and Methods

### 2.1. Participants

A total of 48 male collegiate athletes with patellofemoral pain (PFP) were recruited from Xi’an Physical Education University and randomly assigned to either the intervention group (*n* = 24) or the control group (*n* = 24). All participants maintained their usual sport-specific training schedules throughout the study period. The diagnosis of PFP was confirmed by a certified rehabilitation therapist with experience in sports injury assessment according to predefined criteria based on international consensus recommendations for PFP [19]. Regarding symptom laterality, participants with either unilateral or bilateral PFP were eligible. For participants with bilateral PFP, the limb with greater baseline pain intensity was selected as the tested limb for all limb-specific assessments, including hip and ankle mobility, sEMG testing, and dynamic balance assessment. Participants were eligible if they had recurrent peripatellar or retropatellar pain during the previous week, symptom duration of at least 4 weeks, pain provoked by at least one loaded knee-flexion activity, and reproducible peripatellar or retropatellar pain during squatting. The inclusion criteria were as follows: (1) male collegiate athletes aged 18–25 years; (2) at least 3 years of systematic sport-specific training experience and currently participating in sport-specific training at least three times per week; (3) recurrent peripatellar or retropatellar pain consistent with PFP during the previous week; (4) pain provoked by at least one loaded knee-flexion activity, including squatting, stair ascent or descent, running, jumping, or prolonged sitting, with reproducible peripatellar or retropatellar pain during squatting; (5) symptom duration of at least 4 weeks; and (6) the ability to complete all testing and intervention procedures. The exclusion criteria were as follows: (1) a history of knee surgery or major lower-limb surgery; (2) acute lower-limb injury within the previous 6 months; (3) other clearly diagnosed conditions that may cause anterior knee pain, including ligament injury, meniscal injury, patellar instability, osteoarthritis, or other tibiofemoral joint disorders; (4) neurological or systemic diseases that could affect physical performance; and (5) current participation in other structured lower-limb rehabilitation programs.

### 2.2. Ethical Approval

This study was conducted in accordance with the principles of the Declaration of Helsinki and was approved by the Ethics Committee of Xi’an Physical Education University (approval number: XAIPE202585; approval date: 31 December 2025). All participants provided written informed consent before taking part in the study.

### 2.3. Trial Registration

This trial was retrospectively registered at ClinicalTrials.gov (NCT07542236; protocol ID: XAIPE-PFP-2025-01; first posted: 14 April 2026). The ethics-approved study protocol, including the predefined co-primary outcomes, secondary outcomes, intervention procedures, testing procedures, and planned statistical analyses, was approved by the Ethics Committee of Xi’an Physical Education University on 31 December 2025 (approval number: XAIPE202585), before participant recruitment began. The study started on 15 January 2026, the primary completion date was 2 March 2026, and the study completion date was 4 March 2026. Therefore, trial registration occurred after participant recruitment and after completion of data collection.

The statistical analysis plan was included in the ethics-approved study protocol and research records before formal data analysis. However, because the trial was not prospectively registered, these prespecified procedures were not independently verifiable through a public trial-registration record before participant enrollment. Accordingly, retrospective registration remains an important methodological limitation. To reduce concerns regarding selective outcome reporting, all outcomes described in the Section 2 were analyzed and reported in the Section 3.

### 2.4. Study Design

**Figure 1:**
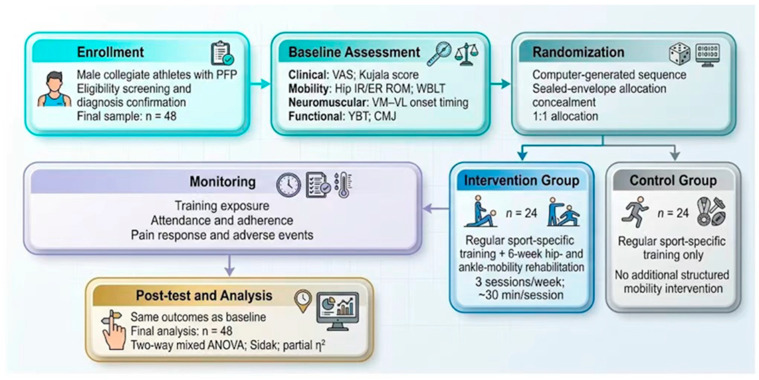
Experimental design of the PFP rehabilitation study in male collegiate athletes. Different colors are used to distinguish the main study stages, including enrollment, baseline assessment, randomization, monitoring, intervention allocation, and post-test analysis; the colors do not represent statistical categories or outcome differences.

This study used an assessor-blinded, randomized controlled, pre–post, parallel-group design to investigate the effects of a hip and ankle mobility-based rehabilitation program on pain, neuromuscular function, and lower-limb functional performance in male collegiate athletes with PFP. Participant recruitment, baseline assessment, intervention implementation, and post-intervention assessment were conducted at Xi’an Physical Education University between 15 January 2026 and 4 March 2026. After completing baseline assessments, participants were randomly assigned to either the intervention group or the control group in a 1:1 ratio using a computer-generated randomization sequence generated in Microsoft Excel (Microsoft Corporation, Redmond, WA, USA). The randomization sequence was generated by an independent researcher who was not involved in participant recruitment, intervention delivery, outcome assessment, or data analysis. Allocation concealment was implemented using sequentially numbered, opaque, sealed envelopes. Each envelope was opened only after completion of baseline testing by a researcher who was not involved in outcome assessment. The randomization sequence was generated by an independent researcher who was not involved in participant recruitment, intervention delivery, outcome assessment, or data analysis. Allocation concealment was implemented using sequentially numbered, opaque, sealed envelopes. Each envelope was opened only after completion of baseline testing by a researcher who was not involved in outcome assessment. The intervention lasted 6 weeks, and all participants completed post-intervention assessments (Post) after the intervention period. Owing to the nature of the intervention, blinding of participants and rehabilitation personnel was not feasible. However, outcome assessors remained blinded to group allocation throughout the study. To enhance internal validity, pre- and post-intervention assessments were conducted using the same testing procedures, equipment, testing order, and time window for each participant. The overall experimental design, including randomization, intervention allocation, monitoring procedures, and post-intervention analysis, is shown in Figure 1. The reporting of this randomized controlled trial followed the CONSORT reporting principles. A completed CONSORT checklist is provided as an accompanying reporting document.

### 2.5. Hip and Ankle Mobility-Based Rehabilitation Program

Participants in the intervention group completed a 6-week hip and ankle mobility-based rehabilitation program, consisting of three sessions per week, with each session lasting approximately 30 min. The program was designed based on the lower-limb kinetic-chain concept and the proximal–distal interaction model in PFP rehabilitation. Its primary aim was to address hip rotational mobility restrictions and limited weight-bearing ankle dorsiflexion, which may contribute to increased patellofemoral joint loading, and to facilitate the transfer of mobility gains into functional lower-limb movement control.

The 6-week duration and session dosage were selected to balance rehabilitation adaptation and practical feasibility in collegiate athletes who continued regular sport-specific training. Specifically, the 6-week period allowed the program to be organized into three progressive 2-week phases, including basic mobility restoration, expansion of available range of motion and end-range control, and functional transfer to sport-related lower-limb movement patterns. The frequency of three sessions per week was chosen to provide repeated therapeutic exposure and sufficient practice opportunities for mobility and movement-control tasks, while limiting excessive interference with participants’ usual sport-specific training schedules. Each session lasted approximately 30 min, which allowed sufficient time to complete the standardized warm-up, hip mobility training, ankle mobility training, and integrated movement exercises within a feasible and acceptable rehabilitation format. A shorter 4-week intervention may have been insufficient to observe meaningful transfer from mobility improvement to neuromuscular coordination and functional movement outcomes, whereas a longer 8-week intervention could have increased participant burden and reduced adherence. The intervention was supervised on site by trained rehabilitation personnel, with an approximate supervision ratio of one rehabilitation staff member to 4–6 participants. Before the intervention began, all rehabilitation staff received standardized training regarding the exercise procedures, movement criteria, pain-monitoring rules, progression criteria, and safety precautions to ensure consistency of intervention delivery across participants. Each session was conducted in a fixed training environment. During each session, the rehabilitation staff monitored movement quality according to predefined criteria and recorded exercise completion, pain responses, and any required modifications [20].

Each rehabilitation session consisted of four components: (1) standardized warm-up; (2) hip mobility training; (3) ankle mobility training; and (4) integrated movement exercises. The standardized warm-up was used to prepare participants for exercise and typically included low-intensity aerobic activity and dynamic stretching. Hip mobility training primarily targeted hip internal and external rotation, with an emphasis on improving hip rotational range of motion and end-range control. Ankle mobility training focused on weight-bearing dorsiflexion and was implemented through calf flexibility exercises, closed-chain tibial progression tasks, and progressive knee-to-wall dorsiflexion drills. Integrated movement exercises were used to promote the transfer of hip and ankle mobility gains into lower-limb alignment control, squat-pattern mechanics, and sport-related movement control [21].

The intervention was divided into three consecutive phases, each lasting 2 weeks. During weeks 1–2, the primary goals were to restore basic hip and ankle mobility, improve movement awareness, and establish pain-free or low-pain movement patterns. During weeks 3–4, the focus shifted toward increasing available range of motion, improving end-range control, and enhancing closed-chain ankle dorsiflexion function. During weeks 5–6, the emphasis was placed on transferring mobility improvements into more functional lower-limb movement patterns and refining task-specific control relevant to sport participation.

To improve reproducibility and intervention reporting in accordance with TIDieR principles, the main intervention table was expanded to include exercise names, session components, dosage, repetitions or hold duration, intensity regulation, ROM targets, progression criteria, regression criteria, supervision ratio, and adherence monitoring procedures. Exercise dose, intensity, ROM targets, progression criteria, supervision details, and adherence monitoring procedures are summarized in Table 1.

Participants in the control group maintained their regular sport-specific training during the same 6-week period but did not receive any additional structured hip or ankle mobility intervention. This design was selected because the primary aim was to determine whether adding a hip and ankle mobility-based rehabilitation program to usual sport-specific training would provide additional benefits compared with usual sport-specific training alone. No active, sham, or attention-matched activity was provided to the control group. To minimize intervention contamination, all participants were instructed to maintain their usual sport-specific training routines and not to initiate any additional structured lower-limb rehabilitation, mobility training, or new resistance-training program during the study period. Sport-specific training exposure was monitored weekly using self-report logs, including training frequency, approximate training duration, and whether any major changes in training content or training load occurred. These logs were reviewed by the research team to identify potential deviations from usual training exposure during the intervention period.

### 2.6. Training Load and Daily Activity Control

Throughout the study period, participants in both groups were instructed to maintain their usual sport-specific training schedules and daily lifestyle habits. Apart from the content specified in the study protocol, participants were not permitted to perform any additional structured lower-limb rehabilitation, mobility training, or resistance training. Participants were instructed to maintain their habitual diet and lifestyle habits throughout the study period and not to initiate any new nutritional supplements. They were also instructed to avoid analgesic or anti-inflammatory medication before official testing unless medically necessary. Any medication use, supplement use, or unusual health condition during the intervention period was reported to the research staff and recorded when applicable. Weekly sport-specific training frequency and total weekly training duration were recorded during the study period to monitor whether regular training exposure remained stable between groups, consistent with recommendations that athlete training load should be monitored when interpreting adaptations and performance-related outcomes [22]. To minimize the potential influence of acute fatigue on outcome measures, all participants were instructed to avoid high-intensity exercise within 24 h before each formal testing session [23].

### 2.7. Testing Procedures and Standardization

Pre-intervention (Pre) and post-intervention (Post) assessments followed the same standardized testing procedures. To reduce the potential influence of circadian variation on neuromuscular performance, the pre- and post-intervention assessments for each participant were conducted within the same time window (±1 h) [24]. Before formal testing, all participants completed a standardized warm-up consisting of 5 min of jogging, dynamic stretching, and task-specific preparatory activities.

To minimize testing-order effects and fatigue accumulation, all outcome measures were assessed in a fixed order, with standardized recovery intervals between tests. The testing sequence was as follows: (1) pain assessment and knee-related function questionnaire; (2) hip and ankle mobility assessments; (3) surface electromyography during the double-leg squat task; (4) Y-Balance Test; and (5) countermovement jump (CMJ) test. Before baseline testing, all participants completed one familiarization session to improve task familiarity and measurement stability.

To improve measurement consistency, all assessors were trained before data collection, and the same standardized procedures, equipment, testing order, recovery intervals, and testing time window were used at both Pre and Post assessments. For outcomes requiring repeated trials, three valid trials were performed, and the mean or best value was used according to the predefined protocol. The selected outcome measures and procedures were based on previous evidence supporting their reliability or standardized use, including the Kujala score, hip rotation range of motion, weight-bearing lunge test, Y-Balance Test, and force-plate-based CMJ assessment. For VM–VL onset timing, electrode placement, signal acquisition, and preprocessing procedures followed established surface electromyography recommendations. However, a separate test–retest reliability analysis was not conducted in the present sample before the intervention.

For all unilateral outcome measures, the tested limb was determined using a standardized rule. For participants with unilateral PFP, the affected limb was defined as the painful limb. For participants with bilateral PFP, the limb with the higher baseline VAS score was selected as the tested limb. If pain severity was equal between limbs, the dominant limb was selected.

### 2.8. Outcomes

The co-primary clinical outcomes were pain intensity and knee-related function, assessed using a 10-cm visual analog scale (VAS) and the Kujala Anterior Knee Pain Scale, respectively. The VAS was used to reflect changes in PFP-related pain symptoms, whereas the Kujala score was used to assess PFP-related functional limitations and daily or sport-related activity capacity. These two measures represented the symptom and function domains, respectively, and were used jointly to evaluate the primary clinical effects of the intervention.

Secondary outcomes included hip internal and external rotation range of motion, weight-bearing ankle dorsiflexion, VM–VL relative onset timing, Y-Balance Test composite score, and countermovement jump height. These secondary outcomes were used to describe targeted mobility changes, exploratory neuromuscular-timing responses, and exploratory functional transfer, and were not independently powered as confirmatory outcomes. Hip and ankle mobility outcomes were used to assess the direct biomechanical targets of the intervention. VM–VL relative onset timing was included as an exploratory neuromuscular-timing outcome during a standardized double-leg squat task. This outcome was used to describe changes in relative quadriceps activation timing, but it was not intended to serve as a direct indicator of patellar tracking, knee joint loading, or clinical recovery. The Y-Balance Test and countermovement jump height were used to evaluate whether mobility improvements were further transferred to lower-limb functional performance.

#### 2.8.1. VAS Pain Intensity Assessment

Pain intensity was assessed using a 10-cm visual analog scale (VAS). Participants reported their average anterior knee pain associated with PFP-related provocative activities during the previous week. A score of 0 indicated “no pain”, whereas a score of 10 indicated “worst imaginable pain” [25,26].

#### 2.8.2. Kujala Knee-Related Function Assessment

Knee-related function was assessed using the Kujala Anterior Knee Pain Scale. This scale is specifically designed to evaluate patellofemoral-related symptoms and functional limitations during daily and physical activities. Higher scores indicate better knee-related function [27].

#### 2.8.3. Hip and Ankle Range of Motion

Hip and ankle range of motion (ROM) were assessed as secondary outcomes. Hip mobility assessment focused on internal and external rotation, whereas ankle mobility assessment focused on weight-bearing dorsiflexion.

Hip internal and external rotation ROM were measured using a standard goniometer. During testing, the hip and knee of the tested limb were positioned at approximately 90° of flexion. The contralateral limb remained relaxed, and the assessor stabilized the pelvis and femur as needed to minimize compensatory movement. This standardized position was used because hip rotation ROM measurements can differ by testing position, and supine hip rotation assessment has shown favorable reliability compared with other positions [28]. Three valid measurements were recorded for each variable, and the mean value was used for statistical analysis.

**Figure 2:**
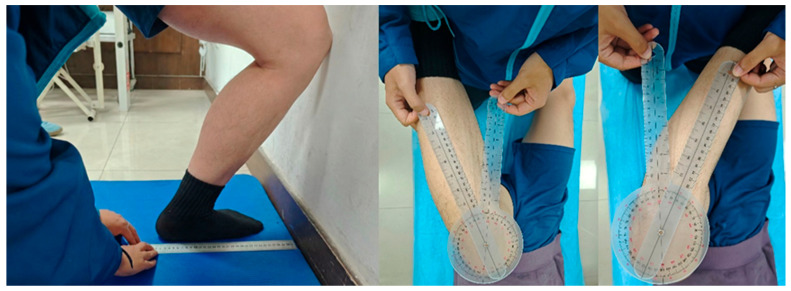
Hip and ankle mobility assessments. From left to right: weight-bearing lunge test, hip internal rotation range-of-motion assessment, and hip external rotation range-of-motion assessment. ROM, range of motion; WBLT, weight-bearing lunge test.

Weight-bearing ankle dorsiflexion ROM was assessed using the weight-bearing lunge test (WBLT). Participants stood facing a wall, with the tested limb placed in front. While keeping the heel in contact with the ground, participants advanced the knee toward the wall in line with the second toe. The foot was progressively moved backward until the maximum toe-to-wall distance at which the knee could still touch the wall without heel lift was identified. This maximum distance was recorded in centimeters. Three valid trials were performed, and the mean value was used for analysis [29,30]. Representative images of the hip and ankle mobility assessments are shown in Figure 2.

#### 2.8.4. VM–VL Onset Timing

**Figure 3:**
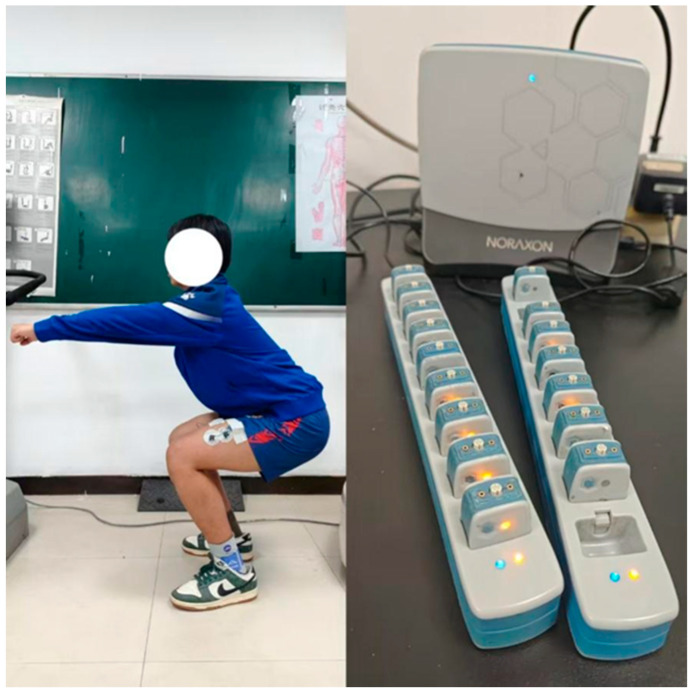
Surface electromyography setup for VM–VL onset timing assessment. (**Left**) standardized double-leg squat task with surface electrodes placed over the vastus medialis and vastus lateralis; (**Right**) wireless surface electromyography system. VM, vastus medialis; VL, vastus lateralis.

Surface electromyography (sEMG) signals of the vastus medialis (VM) and vastus lateralis (VL) on the affected limb were recorded using a wireless sEMG system (Noraxon Ultium (Noraxon USA Inc., Scottsdale, AZ, USA)). Signal acquisition and management were performed using Noraxon MR3 software (version 3.12.70; Noraxon USA Inc., Scottsdale, AZ, USA). Before testing, the skin over the affected thigh was prepared using standard procedures, including shaving when necessary, light abrasion, and alcohol cleaning to reduce skin impedance. Disposable bipolar Ag/AgCl surface electrodes were then placed over the muscle bellies of the VM and VL according to SENIAM recommendations, with the electrode orientation aligned as closely as possible with the direction of the muscle fibers. The inter-electrode distance was set at 20 mm. The reference electrode was placed over a bony area on the anteromedial aspect of the ipsilateral tibia. Electrode placement, acquisition procedures, and testing conditions were kept consistent between pre- and post-intervention assessments [31,32]. The sEMG setup and standardized double-leg squat task are shown in Figure 3.

The sEMG task was a standardized double-leg squat. Participants stood with their feet shoulder-width apart and arms extended forward. Under a standardized verbal command, they performed a controlled squat to approximately 70° of knee flexion and then returned to the starting position. Squat depth was monitored by the assessor during testing to maintain consistency across trials. Before formal testing, all participants completed familiarization trials to reduce learning effects. Movement quality was continuously monitored during testing to minimize trial-to-trial variability. Each participant completed three valid double-leg squat trials, with 60 s of rest between trials. Trials with clear movement deviation, signal artifacts, or electrode detachment were considered invalid and repeated. The mean VM–VL relative onset timing from three valid trials was used for statistical analysis.

The standardized double-leg squat was selected because it is a controlled closed-chain task that commonly increases patellofemoral joint loading and can reproduce PFP-related symptoms under standardized laboratory conditions. Compared with more dynamic tasks such as stair descent, step-down, or running, the double-leg squat allowed better control of foot position, squat depth, movement execution, and inter-trial consistency, while reducing excessive movement artifacts during sEMG recording. Therefore, this task was considered appropriate for comparing pre- and post-intervention VM–VL relative onset timing under a reproducible loading condition. Nevertheless, more dynamic and sport-specific tasks may provide additional ecological information and should be considered in future studies.

The sEMG signals were sampled at 2000 Hz and preprocessed using a 20–450 Hz band-pass filter and a 50 Hz notch filter. The filtered signals were full-wave-rectified before onset timing analysis. Muscle onset was determined using a predefined threshold-based method. Specifically, onset time was defined as the point at which the processed EMG signal exceeded the resting baseline mean by three standard deviations for at least 25 ms. The 3-SD threshold was selected to reduce the risk of false-positive onset detection caused by baseline noise or small signal fluctuations, whereas the 25-ms duration criterion was used to avoid classifying brief transient spikes as true muscle activation while retaining sufficient temporal sensitivity for relative onset timing analysis. Similar threshold-based approaches have been widely used in EMG onset detection studies [30]. The same onset detection criterion was applied consistently across all participants, trials, muscles, and testing sessions. VM–VL relative onset timing was then calculated for subsequent analysis. Positive values indicated delayed VM activation relative to VL activation, whereas values closer to 0 indicated more synchronous activation between the two muscles.

#### 2.8.5. Y-Balance Test

**Figure 4:**
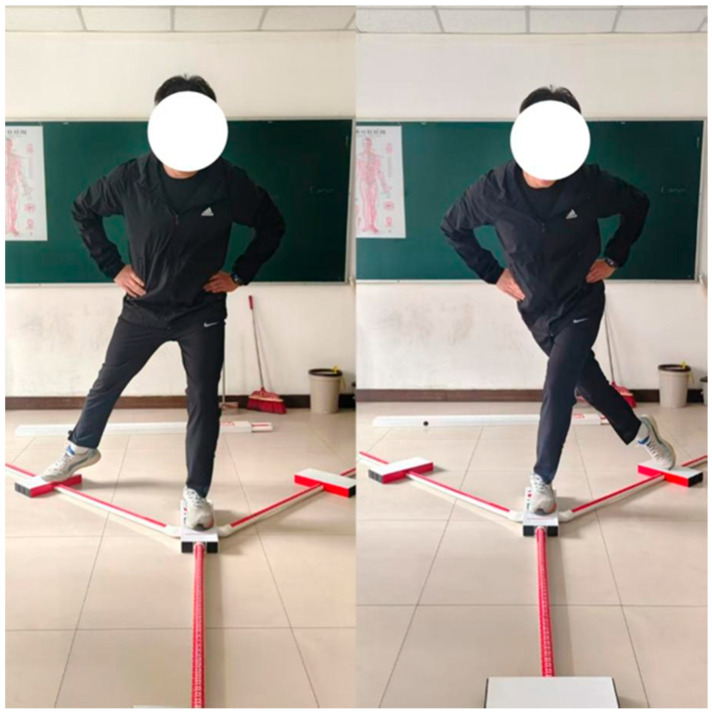
Y-Balance Test assessment. Representative images of the anterior and posterolateral/posteromedial reaching tasks. YBT, Y-Balance Test.

Dynamic balance was assessed using the Y-Balance Test (YBT). During the test, participants stood on one leg and performed maximal reach tasks in the anterior, posteromedial, and posterolateral directions. Before formal testing, all participants completed a standardized warm-up and familiarization trials [33]. Representative images of the Y-Balance Test are shown in Figure 4.

Lower-limb length was measured in the supine position using a measuring tape, from the anterior superior iliac spine to the most distal aspect of the medial malleolus, and was used to normalize reach distances. The affected limb was used as the stance limb for analysis. Three successful trials were completed in each direction, and the mean value was used for analysis. The composite score was calculated using the following formula [34]:

$$\mathrm{Composite}\mathrm{score}\left(\%\right)=[\frac{\mathrm{anterior}+\mathrm{posteromedial}+\mathrm{posterolateral}\mathrm{reach}\mathrm{distances}}{3\times \mathrm{limb}\mathrm{length}}]\times 100$$

The YBT composite score was selected as the main dynamic balance outcome in the present analysis because it provides an overall normalized index of multi-directional reach performance. The anterior, posteromedial, and posterolateral reach distances were recorded to calculate the composite score, but they were not analyzed as separate outcomes in order to maintain a focused outcome framework and reduce unnecessary multiple comparisons.

A trial was considered invalid and repeated if the participant moved the stance foot, used the non-stance foot for support, failed to return to the stable starting position, or did not complete the task as required.

#### 2.8.6. Countermovement Jump

Lower-limb functional performance was assessed using the countermovement jump (CMJ) test, which was performed on a Kistler force plate system (Kistler Instrumente AG, Winterthur, Switzerland). Participants stood in the center of the force plate with their feet shoulder-width apart and hands placed on the hips. They were instructed to perform a rapid downward countermovement followed immediately by a maximal vertical jump. No run-up or visible pause before take-off was permitted.

**Figure 5:**
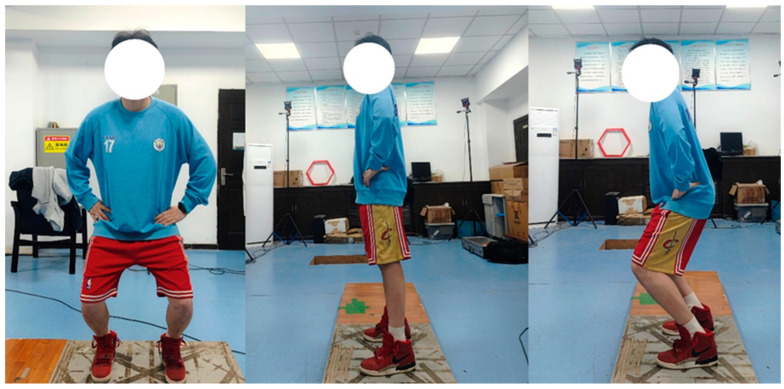
Countermovement jump assessment. From left to right, representative images show the frontal starting position, lateral upright standing position, and countermovement phase before take-off during the standardized CMJ task performed on the force plate. CMJ, countermovement jump.

Before formal testing, all participants completed a standardized warm-up and familiarization jumps. Each participant performed three valid CMJ trials, with 60–90 s of standardized passive recovery between trials to minimize fatigue. Trials were considered invalid and repeated if there were clear movement errors, including an intentional pause before take-off, loss of balance on landing, or failure to follow the standardized movement requirements. The highest jump height from the three trials was used for statistical analysis [35,36]. Representative images of the standardized CMJ assessment are shown in Figure 5.

Vertical ground reaction force (vGRF) data were synchronously collected and exported using XINGYING Post Process software (version 4.4.0.6556; NOKOV Science & Technology Co., Ltd., Beijing, China) at a sampling frequency of 1000 Hz. Jump height was estimated using the take-off velocity method based on force-plate data. Specifically, body weight was first subtracted from the recorded vGRF signal to obtain the net vertical force (Fnet). Fnet was then divided by body mass to calculate instantaneous acceleration, and the vertical velocity of the body center of mass (COM) was obtained by time integration. Take-off was defined as the first sampling point at which vGRF fell below 20 N [37,38]. The vertical take-off velocity (*v_take-off_*) at this point was extracted, and jump height was calculated using the following equation:

$$h=\frac{v_{take-off}^{2}}{2g}$$

where *g* = 9.81 m·s^−2^.

### 2.9. Statistical Analysis

An a priori sample size calculation was performed using G*Power software (version 3.1; Heinrich Heine University Düsseldorf, Düsseldorf, Germany). The sample size estimation was based on the VAS pain intensity outcome within the co-primary clinical outcomes. A medium effect size was assumed (Cohen’s f = 0.25), with a significance level of α = 0.05 and statistical power of 1 − β = 0.80. In GPower, the following options were selected: F tests, ANOVA: repeated measures, within–between interaction. The number of groups was set to 2, the number of measurements to 2, the correlation among repeated measures to 0.50, and the nonsphericity correction ε to 1.00. A total of 48 participants were ultimately included, with 24 participants in each group, satisfying the predefined power requirement [39]. Because the a priori sample-size calculation was based on the VAS pain intensity outcome within the co-primary clinical outcomes, the study was not specifically powered to detect changes in secondary outcomes, including hip rotation range of motion, weight-bearing ankle dorsiflexion, VM–VL relative onset timing, YBT composite score, or CMJ height. Therefore, all secondary outcomes were interpreted as exploratory outcomes. Significant findings for secondary outcomes should be interpreted cautiously because no separate power calculation was performed for these variables, and non-significant findings should not be interpreted as definitive evidence of no effect. Because multiple secondary outcomes were analyzed, the risk of Type I error inflation was acknowledged. No global multiplicity adjustment, such as Holm–Bonferroni correction, was applied across all secondary outcomes because these outcomes were predefined but interpreted as exploratory and hypothesis-generating rather than confirmatory. Therefore, statistical significance for secondary outcomes should be interpreted cautiously and in conjunction with effect sizes, confidence intervals, clinical relevance, and the exploratory nature of these analyses.

All statistical analyses and graph generation were performed using GraphPad Prism 9 (GraphPad Software, Boston, MA, USA). Data are presented as mean ± standard deviation (SD). All statistical tests were two-tailed, and the level of statistical significance was set at *p* < 0.05.

Before parametric analyses, the Shapiro–Wilk test was used to assess normality, and Levene’s test was used to assess homogeneity of variance. Because the within-subject factor of time included only two levels, namely, Pre and Post, the assumption of sphericity and related corrections were not applicable.

For the co-primary clinical outcomes, VAS and Kujala score, as well as all secondary outcomes, a two-way mixed-design ANOVA was used to examine the main effects of group, the main effects of time, and group × time interactions. The group × time interaction was used to determine whether the pattern of change over time differed between the two groups. VAS and Kujala scores were used to evaluate the intervention effects from the symptom and knee-related function domains, respectively. The intervention was considered to show consistent primary clinical benefit only when both co-primary clinical outcomes demonstrated significant group × time interactions in the same favorable direction. The remaining secondary outcomes were used mainly to explore potential biomechanical, neuromuscular, and functional transfer mechanisms and were interpreted cautiously as exploratory outcomes.

When a significant group × time interaction was detected, simple-effects analyses were performed, followed by Sidak-corrected post hoc comparisons to identify the source of the difference. For outcomes without a significant interaction effect, within-group pre–post changes were not used as the primary basis for inference; instead, interpretation was based on the main effects, direction of mean changes, and effect sizes.

Effect sizes for ANOVA results were reported as partial eta squared (partial η^2^). According to commonly used interpretation thresholds, partial η^2^ values of approximately 0.01, 0.06, and 0.14 were considered small, medium, and large effects, respectively. Where appropriate, mean differences and 95% confidence intervals (95% CIs) were reported to improve the completeness of result interpretation. For the co-primary clinical outcomes, within-group changes and between-group differences in change were reported with 95% confidence intervals to enhance clinical interpretability. The clinical relevance of changes in VAS pain intensity and Kujala score was interpreted in relation to commonly reported minimal clinically important difference (MCID) values. For the 10-cm VAS, an improvement of approximately 2 cm has been reported as clinically important, whereas for the Kujala/Anterior Knee Pain Scale, an improvement of approximately 8–10 points has been used as a useful reference range for clinical interpretation. Because MCID estimates may vary according to population, symptom severity, follow-up duration, and calculation method, these values were used as interpretive references rather than absolute thresholds [40].

Baseline demographic and clinical characteristics were summarized using descriptive statistics to assess group comparability before the intervention. Baseline between-group comparisons were performed for descriptive purposes using independent-samples *t*-tests for continuous variables and chi-square tests or Fisher’s exact tests for categorical variables, as appropriate. Because all participants completed the study and were included in the final analysis, no missing-value imputation was performed. All randomized participants were analyzed according to their original group allocation.

## 3. Results

### 3.1. Participant Flow, Adherence, and Baseline Characteristics

**Figure 6:**
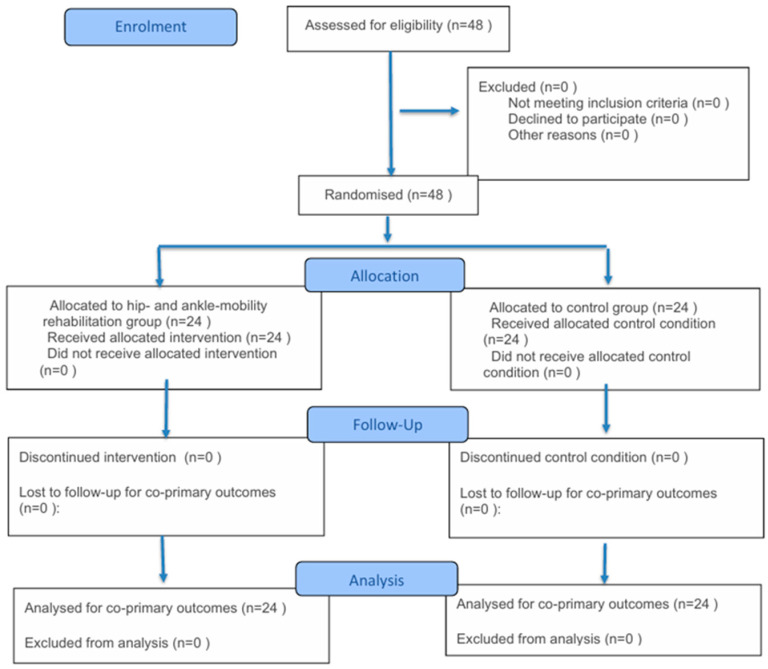
CONSORT 2025 flow diagram of participant enrollment, allocation, follow-up, and analysis.

A total of 48 male collegiate athletes with PFP were recruited and randomized to either the intervention group (*n* = 24) or the control group (*n* = 24). All participants completed the study and were included in the final analysis. The mean attendance rate for supervised rehabilitation sessions in the intervention group was 94% (17/18 sessions). No adverse events or symptom aggravation requiring discontinuation of the intervention were observed during the study period. The CONSORT 2025 flow diagram of participant enrollment, allocation, follow-up, and analysis is shown in Figure 6.

All randomized participants completed the post-intervention assessment and were included in the final analysis population. No participant withdrew from the study. No protocol deviations or intervention-related adverse events were recorded during the study period.

The two groups were generally comparable at baseline in terms of demographic characteristics, body composition, training exposure, PFP symptom duration, and co-primary clinical outcomes. Although sport discipline distribution differed to some extent between groups, both groups included athletes from soccer, volleyball, basketball, and track and field (Table 2).

Regarding symptom laterality, 42 participants had unilateral PFP and 6 participants had bilateral PFP. For participants with bilateral symptoms, the limb with greater baseline pain intensity was selected as the tested limb.

### 3.2. Co-Primary Clinical Outcomes

#### 3.2.1. Pain Intensity

**Figure 7:**
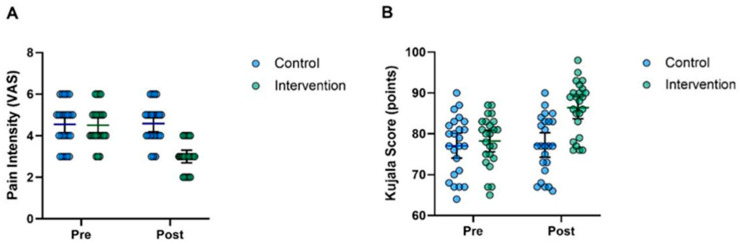
Co-primary clinical outcomes at pre- and post-intervention: (**A**) pain intensity (VAS); (**B**) Kujala score. Data are presented as mean ± SD. Group × time interaction: VAS, *p* < 0.0001, partial η^2^ = 0.436; Kujala score, *p* < 0.0001, partial η^2^ = 0.285.

A significant group × time interaction was observed for pain intensity (*F*(1, 46) = 35.50, *p* < 0.0001, partial η^2^ = 0.436; Figure 7A). Sidak-corrected post hoc comparisons showed no significant pre–post change in VAS score in the control group (mean difference = −0.04, 95% CI: −0.46 to 0.38, *p* = 0.9679), whereas the intervention group showed a significant reduction after the intervention (mean difference = 1.50, 95% CI: 1.08 to 1.92, *p* < 0.0001). The mean VAS score changed from 4.54 to 4.58 in the control group and decreased from 4.50 to 3.00 in the intervention group. The between-group difference in change was −1.54 cm, indicating a greater reduction in pain intensity in the intervention group. This value approached the commonly reported MCID reference of approximately 2 cm for pain improvement on a 10-cm VAS and may therefore be clinically relevant, although it should be interpreted cautiously.

#### 3.2.2. Knee-Related Function

A significant group × time interaction was observed for knee-related function (*F*(1, 46) = 18.37, *p* < 0.0001, partial η^2^ = 0.285; Figure 7B). Sidak-corrected post hoc comparisons showed no significant pre–post change in Kujala score in the control group (mean difference = −0.17, 95% CI: −3.22 to 2.88, *p* = 0.9900), whereas the intervention group showed a significant increase after the intervention (mean difference = 8.17, 95% CI: 5.12 to 11.22, *p* < 0.0001). The mean Kujala score changed from 77.13 to 77.29 in the control group and increased from 78.25 to 86.42 in the intervention group. The between-group difference in change for Kujala score was approximately 8.00 points, indicating greater improvement in knee-related function in the intervention group. This value was within the lower range of commonly reported MCID reference values for the Kujala/Anterior Knee Pain Scale and suggests a potentially clinically meaningful improvement.

### 3.3. Direct Biomechanical Targets of the Intervention

#### 3.3.1. Hip Internal Rotation Range of Motion

**Figure 8:**
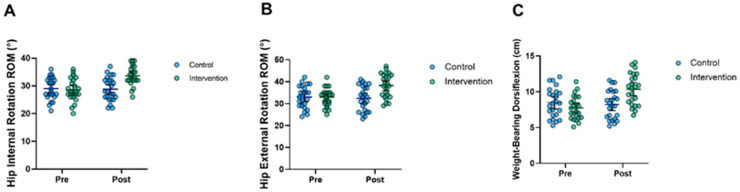
Direct biomechanical outcomes at pre- and post-intervention: (**A**) hip internal rotation range of motion; (**B**) hip external rotation range of motion; (**C**) weight-bearing ankle dorsiflexion. Data are presented as mean ± SD. Group × time interaction: hip internal rotation, *p* < 0.0001, partial η^2^ = 0.332; hip external rotation, *p* < 0.0001, partial η^2^ = 0.342; weight-bearing ankle dorsiflexion, *p* < 0.0001, partial η^2^ = 0.374.

Because secondary outcomes were interpreted as exploratory and no global multiplicity adjustment was performed across all secondary outcomes, these findings should be interpreted cautiously. A significant group × time interaction was observed for hip internal rotation range of motion (*F*(1, 46) = 22.88, *p* < 0.0001, partial η^2^ = 0.332; Figure 8A). Sidak-corrected post hoc comparisons showed no significant pre–post change in the control group (mean difference = 0.21°, 95% CI: −1.64 to 2.06, *p* = 0.9583), whereas the intervention group showed a significant increase after the intervention (mean difference = 5.21°, 95% CI: 3.36 to 7.06, *p* < 0.0001). Mean hip internal rotation range of motion changed from 29.00° to 28.79° in the control group and increased from 28.46° to 33.67° in the intervention group. Although the increase in hip internal rotation was statistically significant and consistent with the predefined mobility target of the intervention, the magnitude of this change should be interpreted cautiously because study-specific measurement error indices, such as the standard error of measurement or minimal detectable change, were not calculated.

#### 3.3.2. Hip External Rotation Range of Motion

A significant group × time interaction was observed for hip external rotation range of motion (*F*(1, 46) = 23.94, *p* < 0.0001, partial η^2^ = 0.342; Figure 8B). Sidak-corrected post hoc comparisons showed no significant pre–post change in the control group (mean difference = 0.58°, 95% CI: −1.39 to 2.56, *p* = 0.7485), whereas the intervention group showed a significant increase after the intervention (mean difference = 5.33°, 95% CI: 3.36 to 7.31, *p* < 0.0001). Mean hip external rotation range of motion changed from 32.96° to 32.38° in the control group and increased from 32.88° to 38.21° in the intervention group. Similarly, the increase in hip external rotation should be interpreted as a statistically significant target response, but its exact clinical magnitude should be considered cautiously because measurement error was not directly quantified.

#### 3.3.3. Weight-Bearing Ankle Dorsiflexion

A significant group × time interaction was observed for weight-bearing ankle dorsiflexion (*F*(1, 46) = 27.51, *p* < 0.0001, partial η^2^ = 0.374; Figure 8C). Sidak-corrected post hoc comparisons showed no significant pre–post change in the control group (mean difference = 0.25 cm, 95% CI: −0.64 to 1.14, *p* = 0.7754), whereas the intervention group showed a significant increase after the intervention (mean difference = 2.61 cm, 95% CI: 1.72 to 3.50, *p* < 0.0001). Mean WBLT distance changed from 8.46 cm to 8.22 cm in the control group and increased from 7.74 cm to 10.35 cm in the intervention group.

### 3.4. Neuromuscular Coordination Outcome

**Figure 9:**
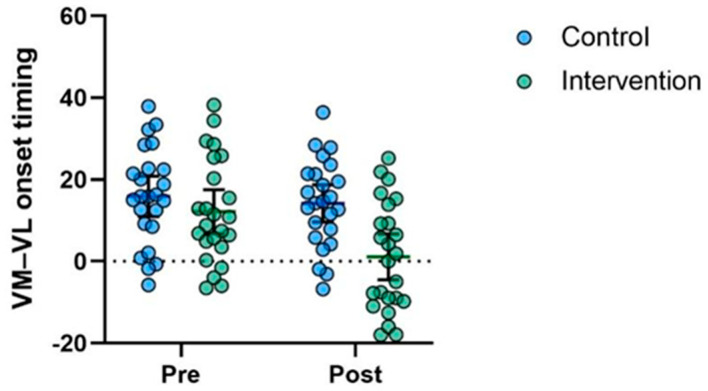
VM–VL onset timing in the control and intervention groups at pre- and post-intervention. Data are presented as mean ± SD. Group × time interaction: *p* = 0.0405, partial η^2^ = 0.088.

A significant group × time interaction was observed for VM–VL relative onset timing (*F*(1, 46) = 4.443, *p* = 0.0405, partial η^2^ = 0.088; Figure 9). Sidak-corrected post hoc comparisons showed no significant pre–post change in the control group (mean difference = 1.73 ms, 95% CI: −5.50 to 8.95, *p* = 0.8268), whereas the intervention group showed a significant reduction after the intervention (mean difference = 11.05 ms, 95% CI: 3.82 to 18.27, *p* = 0.0019). Mean VM–VL relative onset timing changed from 15.90 ms to 14.18 ms in the control group and decreased from 12.14 ms to 1.10 ms in the intervention group. Although this interaction reached statistical significance, the effect size was modest; therefore, the VM–VL onset timing result should be interpreted as an exploratory neuromuscular finding rather than definitive evidence of clinically meaningful neuromuscular normalization.

### 3.5. Functional Transfer Outcomes

#### 3.5.1. Y-Balance Test Composite Score

**Figure 10:**
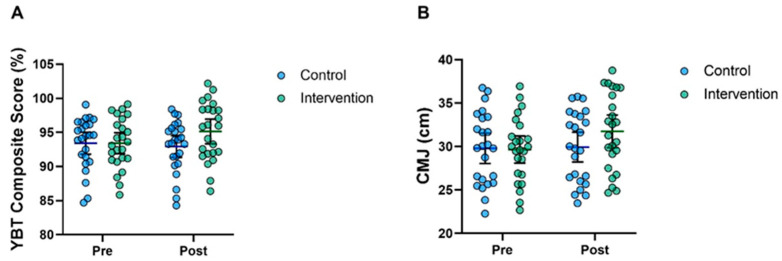
Functional transfer outcomes at pre- and post-intervention: (**A**) Y-Balance Test (YBT) composite score; (**B**) countermovement jump (CMJ) height. Data are presented as mean ± SD. Group × time interaction: YBT composite score, *p* = 0.0144, partial η^2^ = 0.123; CMJ height, *p* = 0.0511, partial η^2^ = 0.080.

A significant group × time interaction was observed for the Y-Balance Test composite score (*F*(1, 46) = 6.472, *p* = 0.0144, partial η^2^ = 0.123; Figure 10A). Sidak-corrected post hoc comparisons showed no significant pre–post change in the control group (mean difference = 0.48, 95% CI: −0.95 to 1.91, *p* = 0.6877), whereas the intervention group showed a significant increase after the intervention (mean difference = 1.74, 95% CI: 0.31 to 3.17, *p* = 0.0140). Mean YBT composite score changed from 93.40% to 92.93% in the control group and increased from 93.41% to 95.15% in the intervention group.

#### 3.5.2. Countermovement Jump Height

No significant group × time interaction was observed for CMJ height (*F*(1, 46) = 4.013, *p* = 0.0511, partial η^2^ = 0.080; Figure 10B). Mean CMJ height changed from 29.78 cm to 29.93 cm in the control group and increased from 29.65 cm to 31.75 cm in the intervention group. Although the intervention group showed a descriptive increase, the interaction effect did not reach the predefined level of statistical significance. Therefore, CMJ height should be interpreted as an exploratory outcome and not as clear evidence of between-group improvement in explosive jump performance.

## 4. Discussion

The main finding of this randomized controlled trial was that, compared with maintaining regular sport-specific training alone, a 6-week hip and ankle mobility-based rehabilitation program produced more favorable changes in pain intensity and knee-related function in male collegiate athletes with PFP. In addition, the intervention improved the predefined biomechanical targets, as reflected by increased hip internal rotation, hip external rotation, and weight-bearing ankle dorsiflexion. The intervention group also showed a reduced VM–VL relative onset timing difference and improved dynamic balance, suggesting that the program may have beneficial effects beyond joint mobility by supporting neuromuscular coordination and partial transfer to lower-limb movement control. However, no significant group × time interaction was observed for CMJ height, indicating that evidence for short-term improvement in explosive jump performance remains limited. Overall, these findings should be interpreted as evidence of short-term changes in clinical symptoms, targeted mobility outcomes, VM–VL onset timing during a controlled squat task, and dynamic balance, rather than as direct evidence of a confirmed biomechanical mechanism. Although the observed improvements in hip rotation range of motion, weight-bearing ankle dorsiflexion, VM–VL onset timing, and YBT composite score are consistent with a possible kinetic-chain-related explanation, this study did not directly measure patellar tracking, knee kinematics, lower-limb joint kinetics, joint moments, joint reaction forces, patellofemoral contact stress, or in vivo patellar motion. Therefore, it cannot be concluded that the intervention improved patellar tracking, normalized knee mechanics, or reduced patellofemoral joint loading. The observed findings demonstrate associations between the supervised mobility-based rehabilitation program and changes in selected clinical, mobility, neuromuscular-timing, and functional outcomes, while the underlying biomechanical mechanism remains hypothetical and requires confirmation in future studies using direct biomechanical and imaging-based assessments.

The magnitude of improvement in the co-primary clinical outcomes should be interpreted by distinguishing statistical significance from clinical meaningfulness. The between-group difference in VAS change was −1.54 cm, which indicates a statistically significant reduction in pain but does not clearly exceed the commonly cited 2-cm MCID threshold for pain intensity on a 10-cm VAS. Therefore, although the pain reduction favored the intervention group, its clinical meaningfulness should be interpreted cautiously and regarded as modest. In contrast, the between-group difference in Kujala score change was 8.00 points, which falls within or near the lower range of commonly reported MCID values for the Kujala/Anterior Knee Pain Scale. This suggests that the improvement in knee-related function may be more clinically convincing than the VAS change. Overall, the findings indicate statistically significant and potentially clinically meaningful short-term improvements, particularly for knee-related function, but they should not be overinterpreted as large or definitive clinical effects. The observed improvements in pain and knee-related function may be cautiously interpreted as being associated with changes in modifiable constraints within the lower-limb kinetic chain, rather than as evidence of a directly measured reduction in patellofemoral joint load [41]. In athletes with PFP, pain provoked during squatting, running, jumping, or stair-related tasks has been linked to altered movement strategies across the hip–knee–ankle complex [42]. When hip rotational mobility or weight-bearing ankle dorsiflexion is limited, athletes may have fewer available movement options for controlling femoral alignment, tibial progression, and lower-limb shock absorption during closed-chain tasks [43]. In the present study, improvements in hip and ankle mobility occurred alongside improvements in pain and knee-related function, which is consistent with a possible kinetic-chain-related explanation. However, because joint moments, joint reaction forces, three-dimensional lower-limb kinematics, joint kinetics, patellofemoral contact stress, and in vivo patellar tracking were not measured, it cannot be concluded that the intervention directly reduced patellofemoral joint loading. Therefore, the clinical improvements should be interpreted as being associated with improved mobility and movement-control-related outcomes, rather than as direct evidence of reduced patellofemoral stress. Relatedly, the absence of movement analysis limits the ability to determine whether the observed mobility gains translated into changes in actual movement strategies during functional tasks. Variables such as squat kinematics, dynamic knee valgus, trunk motion, hip internal rotation during closed-chain movement, landing mechanics, and cutting mechanics were not assessed. Therefore, although the present findings are consistent with a possible kinetic-chain-related explanation, the study cannot confirm whether the intervention altered task-specific lower-limb movement mechanics.

The simultaneous improvements in hip internal rotation, hip external rotation, and weight-bearing ankle dorsiflexion suggest that the intervention affected the predefined proximal and distal mobility targets. This target response is mechanistically relevant because hip and ankle mobility are not isolated anatomical characteristics, but functional constraints that influence movement strategies during closed-chain loading tasks [44]. Improved hip rotational mobility may provide greater adaptability for femoral motion during multiplanar tasks such as squatting, landing, and running, thereby reducing the need for compensatory patterns such as excessive femoral adduction, internal rotation, or dynamic knee valgus. Similarly, improved weight-bearing ankle dorsiflexion may reduce distal restrictions on tibial progression, allowing more effective foot–ankle control and limiting excessive transfer of mechanical demand toward the knee [45]. The combined reduction in proximal and distal constraints may therefore contribute to a more favorable mechanical environment for the patellofemoral joint by improving lower-limb alignment, load distribution, and movement efficiency [46]. However, because this study did not include three-dimensional motion analysis, joint kinetics, or direct assessment of patellofemoral contact stress, it cannot be concluded that the intervention directly altered in vivo patellar tracking [47]. A more cautious interpretation is that improvements in targeted mobility outcomes provide indirect support for a kinetic-chain-based mechanism in PFP rehabilitation.

The reduction in VM–VL relative onset timing should be interpreted cautiously as an exploratory neuromuscular-timing finding observed during a standardized double-leg squat task. Although altered VM–VL timing has been discussed in relation to PFP [48], its clinical relevance remains controversial, and current evidence does not support VM delay as an isolated causal mechanism that fully explains PFP symptoms or patellofemoral joint control [49]. VM–VL onset timing alone cannot confirm improved patellar tracking, reduced patellofemoral joint loading, normalized knee mechanics, or superior clinical recovery. In the present study, the sEMG task was limited to a controlled double-leg squat, which was selected to improve standardization and reduce movement-related sEMG artifacts. However, this task does not represent more dynamic or sport-specific activities such as stair descent, step-down tasks, running, landing, or cutting. In addition, no separate test–retest reliability assessment was performed for VM–VL onset timing in the present sample. PFP is a multifactorial condition, and quadriceps timing should be considered together with hip and ankle mobility, lower-limb alignment, movement strategy, training load, and symptom-related adaptations [50]. Therefore, the observed change should be regarded as an exploratory task-specific neuromuscular-timing response rather than definitive evidence of a clinically meaningful or generalizable neuromuscular adaptation.

The improvement in YBT composite score suggests that the intervention may have produced partial functional transfer to dynamic postural control [51]. However, this interpretation should be limited to the specific functional outcomes assessed in the present study. The YBT reflects dynamic balance and multi-directional reach capacity under single-leg support, and performance requires coordinated control of the hip, knee, and ankle while maintaining lower-limb alignment. These outcomes do not fully capture broader functional movement capacity, such as running mechanics, landing mechanics, cutting maneuvers, sport-specific movement tasks, or activities of daily living. Increased hip rotational mobility and weight-bearing ankle dorsiflexion may therefore have improved participants’ capacity to control the lower limb in weight-bearing end ranges, contributing to enhanced dynamic balance performance. In contrast, CMJ height did not reach the predefined level of statistical significance, which helps define the functional boundary of this mobility-dominant intervention for short-term explosive performance. This finding is consistent with the principle of training specificity, which suggests that training adaptations are strongly influenced by the specific movement pattern, contraction velocity, loading characteristics, and neuromuscular demands of the training stimulus. Because the present intervention primarily targeted hip and ankle mobility and lower-limb movement control rather than resistance, plyometric, or maximal power-development training, the absence of a clear CMJ improvement is not unexpected and should not be interpreted as evidence that the program lacked clinical value. Therefore, CMJ height should be interpreted as an exploratory functional transfer outcome rather than a primary expected adaptation [52].

Thus, the present findings suggest that hip and ankle mobility-based rehabilitation for athletes with PFP should be positioned as a targeted strategy for improving symptoms, movement quality, and dynamic control, rather than as a short-term performance-enhancement intervention for explosive power [53].

These findings should be interpreted within the broader evidence base for PFP rehabilitation. Current clinical recommendations generally support exercise therapy as a core treatment strategy, particularly hip- and knee-focused strengthening, often combined with education, load management, movement retraining, and other adjunctive interventions when clinically indicated [7]. Compared with traditional strengthening-dominant programs, the present intervention primarily targeted hip rotational mobility, weight-bearing ankle dorsiflexion, lower-limb alignment, and movement control. Therefore, the findings should not be interpreted as showing that mobility-based rehabilitation is superior to hip strengthening, combined hip–knee exercise, or multimodal rehabilitation. Rather, this program may represent a targeted adjunctive option for athletes with identifiable proximal and distal mobility restrictions, especially when the clinical goal is to reduce symptoms, restore movement options, and improve early-stage dynamic control without adding substantial fatigue load. Future studies should directly compare mobility-based rehabilitation with hip-focused strengthening, combined hip–knee exercise, and guideline-based multimodal rehabilitation programs to determine their relative and combined effects.

From a clinical practice perspective, this study supports the use of hip and ankle mobility-based rehabilitation as a low-fatigue adjunct to regular sport-specific training in athletes with PFP. The program required only three sessions per week, each lasting approximately 30 min, and no adverse events or symptom aggravation requiring discontinuation were observed during the study. These findings suggest that the program is feasible and safe for collegiate athletes with demanding training schedules. Clinically, this approach may be particularly suitable for early symptom management, restoration of restricted mobility, and early-stage movement-control retraining [6]. It may provide targeted rehabilitation support without substantially increasing overall neuromuscular fatigue load.

Several limitations should be acknowledged. First, the external validity of the present findings is limited. All participants were young male collegiate athletes recruited from a single university, which restricts the generalizability of the results. Therefore, the present findings should not be generalized to female athletes, adolescent athletes, recreational athletes, older adults, non-athletic individuals, or patients with different symptom durations, activity levels, training backgrounds, and clinical presentations. This limitation is clinically important because sex, age, sport participation level, training exposure, movement demands, and symptom history may influence PFP mechanisms, symptom persistence, rehabilitation tolerance, and response to treatment. Accordingly, the findings should be interpreted as applying primarily to young male collegiate athletes with PFP who continued regular sport-specific training during the study period. Future multicenter studies should include more diverse PFP populations to determine whether similar clinical, mobility, neuromuscular-timing, and functional responses can be observed across different sexes, age groups, sport levels, and activity backgrounds [54]. Second, the follow-up period was limited to immediate post-intervention assessment, which represents an important limitation of the present study. No medium- or long-term follow-up assessment was conducted after the 6-week supervised rehabilitation program ended. Therefore, although the intervention was associated with favorable short-term changes in pain, knee-related function, mobility outcomes, VM–VL onset timing, and dynamic balance, it remains unclear whether these improvements were maintained after supervised rehabilitation ceased. In addition, the present study did not assess symptom recurrence, recurrence rates, return-to-sport outcomes, return-to-sport readiness, or maintenance of training participation after the intervention period. This limitation is particularly important because PFP is frequently persistent and recurrent. Future studies should include medium- and long-term follow-up assessments to determine the durability of treatment effects, symptom recurrence, return-to-sport outcomes, and maintenance of mobility and functional improvements [55]. Third, functional transfer was assessed only using the YBT composite score and CMJ height. Although these outcomes reflect dynamic balance and exploratory explosive lower-limb performance, they do not fully represent broader functional movement capacity, including sport-specific movement tasks, running mechanics, landing mechanics, cutting maneuvers, or activities of daily living [56]. Fourth, although changes in joint mobility, sEMG timing, and functional outcomes are consistent with a possible kinetic-chain-related explanation, the present study did not directly measure patellar tracking, joint kinetics, patellofemoral joint loading, patellofemoral contact stress, or in vivo patellar motion. Therefore, the observed reduction in VM–VL relative onset timing should not be interpreted as direct evidence of improved patellar mechanics, normalized knee joint control, or reduced patellofemoral joint stress. In addition, this study did not include two-dimensional or three-dimensional movement analysis; therefore, variables such as squat kinematics, dynamic knee valgus, trunk motion, hip internal rotation during functional tasks, landing mechanics, cutting mechanics, joint moments, and joint reaction forces were not assessed. This limits the ability to determine whether the observed mobility gains translated into measurable changes in task-specific movement strategies or reduced patellofemoral joint loading. Future studies should combine clinical outcomes with three-dimensional motion analysis, joint kinetic assessment, patellofemoral contact stress estimation, or imaging-based patellar tracking evaluation to verify the proposed mechanism. Fifth, the interpretation of the sEMG-derived VM–VL onset timing outcome is limited. VM–VL timing was assessed only during a standardized double-leg squat task, and more dynamic or sport-specific tasks such as stair descent, step-down, running, landing, or cutting were not assessed. Therefore, the findings cannot be generalized to quadriceps activation timing during broader functional or sport-specific movements. In addition, no separate test–retest reliability assessment was performed for VM–VL onset timing or other outcome measures in the present sample. Although standardized procedures, assessor training, familiarization, repeated trials, and blinded assessment were used to reduce measurement error, the absence of study-specific reliability indices weakens confidence in the stability and measurement precision of some observed changes, particularly for neuromuscular-timing outcomes. Future studies should include repeated baseline testing and report reliability indices such as intraclass correlation coefficients, standard error of measurement, and minimal detectable change for the main outcomes. Sixth, the sample-size calculation was based on the VAS pain intensity outcome within the co-primary clinical outcomes and was not designed to provide independent statistical power for the secondary outcomes. Therefore, secondary outcomes, including hip and ankle mobility, VM–VL relative onset timing, YBT composite score, and CMJ height, should be interpreted as exploratory and potentially underpowered. In addition, because multiple secondary outcomes were analyzed and no global multiplicity adjustment was applied across all secondary outcomes, the possibility of Type I error inflation cannot be excluded. Significant findings for secondary outcomes should therefore be interpreted cautiously, and non-significant findings, particularly for CMJ height, should not be interpreted as definitive evidence of no effect. Seventh, the comparator condition was weak and represents an important limitation of the present trial. Participants in the control group continued regular sport-specific training only and did not receive an active, sham, or attention-matched comparator intervention. Therefore, the observed between-group differences may partly reflect non-specific effects related to additional supervised exercise exposure, therapist attention, participant expectations, structured monitoring, placebo-related responses, or Hawthorne effects, rather than mobility-specific mechanisms alone. Accordingly, the findings should be interpreted as the added effect of a supervised hip and ankle mobility-based rehabilitation program compared with regular sport-specific training alone, and not as definitive evidence that mobility restoration itself independently produced the observed improvements. Future studies should include active or attention-matched comparator groups, such as sham mobility exercises, general stretching, general conditioning, strengthening programs, or supervised non-specific exercise sessions matched for contact time, exercise exposure, therapist interaction, and monitoring. Such designs would help isolate the specific therapeutic contribution of hip and ankle mobility restoration. Finally, this trial was retrospectively registered, which represents a major methodological limitation. Although the ethics-approved study protocol, including the planned outcomes and statistical analyses, was approved before participant recruitment began, the registration record was not publicly available before participant enrollment or data collection. Therefore, retrospective registration cannot provide the same level of transparency, independent verification, or protection against outcome switching and selective reporting as prospective registration. This limitation weakens confidence in prospective outcome prespecification and should be considered when interpreting the findings. To improve transparency, we have reported the ethics approval date, study timeline, trial registration date, and the relationship between the ethics-approved protocol and the planned outcomes and analyses in the Section 2. Future trials should be prospectively registered before participant recruitment and should make the trial protocol and statistical analysis plan publicly available before data collection begins [57]. Future studies should include longer follow-up periods, active comparator interventions, more diverse athletic populations, comprehensive functional assessments, and direct biomechanical and movement-analysis measures to further clarify the long-term efficacy, real-world transferability, in vivo mechanisms, and comparative effectiveness of this mobility-based rehabilitation strategy.

## 5. Conclusions

In young male collegiate athletes with PFP, adding a supervised 6-week hip and ankle mobility-based rehabilitation program to regular sport-specific training was associated with more favorable short-term changes in pain intensity, knee-related function, targeted mobility outcomes, VM–VL relative onset timing during a controlled squat task, and dynamic balance compared with regular sport-specific training alone. However, no significant group × time interaction was observed for CMJ height, indicating that this mobility-focused program should not be interpreted as a short-term strategy for improving explosive jump performance. Because the control group did not receive an active or attention-matched intervention, these findings should be interpreted as the added effect of supervised rehabilitation rather than definitive evidence of mobility-specific treatment effects. Given the limited sample of young male collegiate athletes from a single university and the absence of medium- or long-term follow-up, further studies are needed before these findings can be generalized to other populations or long-term clinical outcomes.

## Figures and Tables

**Table 1:** Detailed TIDieR-style description of the 6-week hip and ankle mobility-based rehabilitation program.

Phase	Session Frequency and Supervision	Exercise Component and Exercise Names	Dose and Duration	Intensity and ROM Target	Progression and Regression Criteria
Weeks 1–2	3 sessions/week; approximately 30 min/session; supervised on site by trained rehabilitation personnel; approximate supervision ratio of 1 rehabilitation staff member to 4–6 participants	Standardized warm-up: low-intensity jogging and dynamic lower-limb stretching	5 min	Low intensity; preparation for mobility and movement-control exercises; no increase in anterior knee pain	Proceed to mobility exercises when the participant completed the warm-up without symptom aggravation
Weeks 1–2	Same as above	Hip mobility training: dynamic hip internal and external rotation drills; active-assisted hip rotational stretching	2 sets of 8–10 controlled repetitions for dynamic drills; stretching held for 20 s per set	Symptom-tolerable hip rotational end range; controlled movement without pelvic or trunk compensation	Progress when anterior knee pain was ≤3/10 on the VAS, hip rotation was controlled, and no symptom aggravation occurred within 24 h
Weeks 1–2	Same as above	Ankle mobility training: gastrocnemius and soleus stretching; basic knee-to-wall dorsiflexion drills	2 sets of 8–10 controlled repetitions for dorsiflexion drills; stretching held for 20 s per set	Symptom-tolerable weight-bearing dorsiflexion end range; heel maintained in contact with the ground; knee aligned with the second toe	Progress when dorsiflexion was completed without heel lift, excessive foot pronation, dynamic knee valgus, or increased anterior knee pain
Weeks 1–2	Same as above	Integrated movement exercises: low-load squat-pattern exercises emphasizing hip–knee–ankle alignment	2 sets of 8–10 controlled repetitions	Body mass only; low-to-moderate effort; movement quality prioritized over speed or load	Progress when the squat pattern was completed with stable pelvis, no marked dynamic knee valgus, no heel lift, and pain ≤3/10 on the VAS
Weeks 3–4	3 sessions/week; approximately 30 min/session; supervised on site by trained rehabilitation personnel; approximate supervision ratio of 1:4–6	Hip mobility training: end-range hip rotational control exercises; dynamic hip mobility drills with greater excursion	2–3 sets of 10–12 controlled repetitions; 20–30 s holds when stretching was used	Symptom-tolerable hip rotational end range with active control; no trunk or pelvic compensation	Progress when end-range control was maintained, movement quality remained stable, pain was ≤3/10 on the VAS, and no symptom aggravation occurred within 24 h
Weeks 3–4	Same as above	Ankle mobility training: progressed calf flexibility tasks; split-stance tibial progression drills; controlled squat-based dorsiflexion exercises	2–3 sets of 10–12 controlled repetitions; 20–30 s holds when stretching was used	Progressive weight-bearing ankle dorsiflexion; heel contact maintained; knee aligned with the second toe	Progress when tibial progression increased without heel lift, excessive foot pronation, dynamic knee valgus, or increased anterior knee pain
Weeks 3–4	Same as above	Integrated movement exercises: controlled squat-based mobility-control drills	2–3 sets of 10–12 controlled repetitions	Body mass only; low-to-moderate effort; controlled hip–knee–ankle alignment	Progress when the participant maintained stable lower-limb alignment and completed the prescribed repetitions without symptom increase
Weeks 5–6	3 sessions/week; approximately 30 min/session; supervised on site by trained rehabilitation personnel; approximate supervision ratio of 1:4–6	Hip and ankle mobility-control training: advanced hip mobility-control tasks; progressive weight-bearing dorsiflexion tasks	3 sets of 8–12 controlled repetitions	Symptom-tolerable end range; greater emphasis on active end-range control and movement quality	Progress when the participant completed the task with pain ≤3/10 on the VAS, no symptom aggravation within 24 h, and no compensatory movement
Weeks 5–6	Same as above	Functional integrated movement exercises: functional squat-based dorsiflexion control; integrated movement drills emphasizing lower-limb alignment and movement efficiency	3 sets of 8–12 controlled repetitions; 30–60 s rest between sets	Body mass only; low-to-moderate effort; movement quality prioritized over speed, external load, or fatigue	Task difficulty was progressed or maintained according to pain response, stable lower-limb alignment, absence of dynamic knee valgus, absence of pelvic deviation, and absence of heel lift

Note: Exercise intensity was regulated according to movement quality, perceived discomfort, and anterior knee pain response. No external resistance load was used. Anterior knee pain was not allowed to exceed 3/10 on the VAS, and no symptom aggravation was permitted within 24 h after training. ROM targets were individualized and defined as the maximum symptom-tolerable hip rotational or weight-bearing ankle dorsiflexion range that could be achieved without compensatory movement. If the progression criteria were not met, exercise range of motion, volume, or task difficulty was reduced, or the participant was returned to exercises from the previous phase. VAS, visual analog scale; ROM, range of motion; TIDieR, Template for Intervention Description and Replication.

**Table 2:** Baseline demographic characteristics, body composition, training exposure, PFP symptom characteristics, sport discipline distribution, co-primary clinical outcomes, and baseline between-group *p*-values of the two groups.

Variable	Control (*n* = 24)	Intervention (*n* = 24)	Total (*n* = 48)	*p* Value
Age (years)	22.2 ± 3.5	22.8 ± 3.6	22.5 ± 3.5	0.561
Height (cm)	177.6 ± 6.1	179.4 ± 5.4	178.5 ± 5.8	0.285
Body mass (kg)	72.2 ± 7.5	74.2 ± 6.8	73.2 ± 7.2	0.338
BMI (kg/m^2^)	22.9 ± 1.2	23.1 ± 1.4	23.0 ± 1.3	0.598
Training experience (years)	5.8 ± 2.1	6.6 ± 1.7	6.2 ± 1.9	0.154
Weekly training frequency (sessions/week)	3.5 ± 0.8	3.3 ± 1.1	3.4 ± 1.0	0.475
Weekly training duration (h/week)	7.7 ± 0.9	7.5 ± 1.3	7.6 ± 1.1	0.539
Symptom duration (weeks)	7.0 ± 1.4	7.4 ± 1.2	7.2 ± 1.3	0.293
Baseline VAS (0–10 cm)	4.54 ± 1.06	4.50 ± 0.93	4.52 ± 0.99	0.89
Baseline Kujala score	77.13 ± 7.21	78.25 ± 6.23	77.69 ± 6.71	0.568
Sport discipline, *n* (%)				0.506
Soccer	8 (33.3%)	4 (16.7%)	12 (25.0%)	—
Volleyball	4 (16.7%)	4 (16.7%)	8 (16.7%)	—
Basketball	6 (25.0%)	6 (25.0%)	12 (25.0%)	—
Track and field	6 (25.0%)	10 (41.7%)	16 (33.3%)	—

Note: Data are presented as mean ± SD or *n* (%). Baseline *p* values are presented for descriptive purposes. Continuous variables were compared using independent-samples *t*-tests, and sport discipline distribution was compared using a chi-square test. BMI, body mass index; PFP, patellofemoral pain; VAS, visual analog scale.

## Data Availability

The datasets generated and/or analyzed during the current study are not publicly available due to privacy and ethical restrictions but are available from the corresponding author on reasonable request.
